# Biased efficacy estimates in phase-III dengue vaccine trials due to heterogeneous exposure and differential detectability of primary infections across trial arms

**DOI:** 10.1371/journal.pone.0210041

**Published:** 2019-01-25

**Authors:** Guido España, Cosmina Hogea, Adrienne Guignard, Quirine A. ten Bosch, Amy C. Morrison, David L. Smith, Thomas W. Scott, Alexander Schmidt, T. Alex Perkins

**Affiliations:** 1 Department of Biological Sciences and Eck Institute for Global Health, University of Notre Dame, Notre Dame, IN, United States of America; 2 GlaxoSmithKline, Rockville, MD, United States of America; 3 United States Naval Medical Research Unit No. 6, Lima, Peru; 4 Department of Entomology and Nematology, University of California, Davis, CA, United States of America; 5 Institute for Health Metrics and Evaluation, University of Washington, Seattle, WA, United States of America; Johns Hopkins Bloomberg School of Public Health, UNITED STATES

## Abstract

Vaccine efficacy (VE) estimates are crucial for assessing the suitability of dengue vaccine candidates for public health implementation, but efficacy trials are subject to a known bias to estimate VE toward the null if heterogeneous exposure is not accounted for in the analysis of trial data. In light of many well-characterized sources of heterogeneity in dengue virus (DENV) transmission, our goal was to estimate the potential magnitude of this bias in VE estimates for a hypothetical dengue vaccine. To ensure that we realistically modeled heterogeneous exposure, we simulated city-wide DENV transmission and vaccine trial protocols using an agent-based model calibrated with entomological and epidemiological data from long-term field studies in Iquitos, Peru. By simulating a vaccine with a true VE of 0.8 in 1,000 replicate trials each designed to attain 90% power, we found that conventional methods underestimated VE by as much as 21% due to heterogeneous exposure. Accounting for the number of exposures in the vaccine and placebo arms eliminated this bias completely, and the more realistic option of including a frailty term to model exposure as a random effect reduced this bias partially. We also discovered a distinct bias in VE estimates away from the null due to lower detectability of primary DENV infections among seronegative individuals in the vaccinated group. This difference in detectability resulted from our assumption that primary infections in vaccinees who are seronegative at baseline resemble secondary infections, which experience a shorter window of detectable viremia due to a quicker immune response. This resulted in an artefactual finding that VE estimates for the seronegative group were approximately 1% greater than for the seropositive group. Simulation models of vaccine trials that account for these factors can be used to anticipate the extent of bias in field trials and to aid in their interpretation.

## Introduction

An estimated 390 million people worldwide experience dengue virus (DENV) infections each year, with approximately 96 million of those showing clinically apparent symptoms [1]. Only one dengue vaccine (Dengvaxia, Sanofi Pasteur) has been licensed to date, although safety concerns have limited its rollout [2]. Beyond Dengvaxia, 22 investigational dengue vaccines are at various stages of development, with six of them in clinical trials [3]. Two tetravalent vaccines have advanced to phase-III clinical trials to evaluate vaccine efficacy against symptomatic, virologically-confirmed dengue disease [4,5]. In addition, 14 investigational Zika vaccines are under development, some of which may also advance to late-phase trials [6]. Late-phase vaccine trials for dengue, Zika, and chikungunya—three viruses with a common mosquito vector in *Aedes aegypti*—share a number of common challenges.

A major challenge for vaccine trials for these diseases derives from the observation that their transmission is characterized by extensive spatial and temporal heterogeneity. For example, epidemiological studies of dengue in both rural [7] and urban [8] settings in Thailand showed that transmission tends to occur focally at spatial scales of 1 km or less and can be much more intense at some times than others. Similar observations are being made for Zika and chikungunya [9,10]. Some of the complications posed to vaccine trials by such strong spatial and temporal heterogeneity include achieving balanced randomization with respect to exposure across trial arms and correctly anticipating exposure rates in advance of a trial.

Another complication for vaccine trials for these diseases also derives from heterogeneity in transmission, but at much finer scales. Field studies [11,12] have shown that patterns of *Ae*. *aegypti* blood-feeding are extremely heterogeneous; i.e., most of the mosquito bites are concentrated on a few, while other people are rarely bitten [13]. These heterogeneities apply to individuals within a single household and become compounded even further [14] when layered on top of house-to-house variation in *Ae*. *aegypti* densities [15] and complex patterns of human [16,17] and mosquito [18] movement, which affect human-mosquito contact. This fine-scale variation results in heterogeneity in the number of DENV exposures that trial participants experience, which can lead to bias in estimates of vaccine efficacy for susceptibility (VE_S_) [19] for “leaky” vaccines, which confer partial protection to all vaccinees rather than full protection to a subset [20]. This type of bias applies to VE_S_ estimates derived using survival methods, such as Cox regression models, unless trial participants experience homogeneity in exposure [21,22]. In the presence of heterogeneities in exposure, frailty models have been proposed to reduce bias in VE_S_ estimates [21]. Measuring exposure or collecting data on risk factors can also help in reducing this bias [22].

Because heterogeneity in exposure is known to induce bias in VE_S_ estimates for leaky vaccines and because there is so much heterogeneity in DENV transmission that would be nearly impossible to account for explicitly in a trial, we used a simulation-based approach to assess the extent to which heterogeneous exposure may bias VE_S_ estimates for dengue vaccines. Leveraging detailed historical understanding of DENV transmission in Iquitos, Peru, we used this model to explore plausible outcomes for hypothetical vaccine efficacy trials that were simulated as if they took place at a specific point in time during the past in Iquitos. This exercise is an initial case study on which additional analyses can be developed for other specific settings of interest as data from other vaccine trial sites become available and amenable to modeling.

## Methods

### Trial overview

We designed a hypothetical, individually randomized phase-III trial of a generic dengue vaccine. The aim of the trial was to evaluate the vaccine efficacy for susceptibility (VE_S_) as a measure of the protective effects of vaccination against the first symptomatic, virologically-confirmed case of dengue caused by any DENV serotype during simulated vaccine trials in Iquitos, Peru. A virologically-confirmed dengue case was defined as febrile illness with temperature ≥ 38°C for at least two consecutive days and a positive result from a reverse-transcriptase polymerase chain reaction (RT-PCR) from a blood sample. This endpoint has been used in phase-III dengue vaccine trials and is recommended by the WHO [23]. We chose to simulate the trial in Iquitos because of its long history of epidemiological studies of dengue, which provide a unique opportunity to parameterize and validate detailed models of DENV transmission [16,24–27]. Iquitos is a city of approximately 370,000 inhabitants, located in the Amazon Basin and accessible only by boat or airplane.

We simulated the active phase of our hypothetical dengue vaccine trials comparable to previous and ongoing trials [5,28–32] (Table 1), such that ours consisted of regular contacts with study participants for at least 13 months after completion of a full course of vaccination (two doses, two months apart). Simulated trial participants were randomly assigned to a placebo or vaccine arm in a 1:1 ratio. We assumed that the placebo conferred no protection against clinical endpoints and that the vaccine conferred some level of protection defined as an input of the model and estimated as VE_S_ with the data collected from the trial. In all simulations, we initialized the trial on June 26, 2009. The period thereafter was characterized by a relatively high force of infection of multiple DENV serotypes [27], which would have been ideal for a trial.

**Table 1:** Characteristics of four vaccine trial designs for dengue vaccines, in addition to our virtual trial.

Variable	Sanofi CYD14 [28]	Sanofi CYD 15 [30]	Takeda [5,32]	Butantan [4,31]	Virtual trial
Sample size	10,278	20,875	20,100	16,944	2,324*
Number of sites	12	22	Not specified	Not specified	1
Vaccine:control ratio	2:1	2:1	1:1	2:1	1:1
Power	0.90	0.90	-	-	0.90
Control	Placebo	Placebo	Placebo	Placebo	Placebo
Target efficacy	0.70	-	-	0.80	0.80
Lower bound of vaccine efficacy	0.25	0.25	-	0.25	0.30
Age groups	2–5,6–11, 12–14	9–11, 12–16	4–16	2–6, 7–17,18–59	5–17, 18–45
Primary endpoint	Symptomatic virologically-confirmed dengue disease	Symptomatic virologically-confirmed dengue disease	Symptomatic virologically-confirmed dengue disease	Symptomatic virologically-confirmed dengue disease	Symptomatic virologically-confirmed dengue disease
Vaccination schedule	Months: 0, 6, 12	Months: 0, 6, 12	Days: 1, 90	Day 1	Days: 1, 60
Expected cases	57	57	120	24	68
Minimum timeframe to estimate VE	1 year	1 year	1 year	1 year	1 year
Statistical analysis	Incidence density	Incidence density	Survival analysis	Incidence density	Survival analysis
Safety follow-up	5 years	5 years	4.75 years	5 years	-
Test for baseline DENV serology	Subset	Subset	Yes	-	Yes

*Baseline scenario

### Vaccine model

We modeled a hypothetical vaccine with a per-exposure protection (PEP) that represents the reduction in risk of disease that the vaccine confers to an individual following a single exposure to DENV. The vaccine reduces the risk of two separate events: successful DENV infection conditional on exposure (RR_inf|exp_) and clinically apparent dengue fever conditional on DENV infection (RR_dis|inf_), such that

$${\text{PEP=1-RR}}_{\text{inf|exp}}^{\rho }{\text{RR}}_{\text{(dis|inf)}}^{1-\rho }\text{,}$$ (1)

where RR_inf|exp_, RR_dis|inf_, and ρ are specified as inputs to the model. Our default assumption was that the vaccine protects equally against infection conditional on exposure and against disease conditional on infection (ρ = 0.5). Our default assumptions about RR_inf|exp_ and RR_dis|inf_ were that they both equaled 0.447, consistent with an exploratory PEP value of 0.8 as assumed in planning for the ongoing Butantan trial [31]. In our model, the vaccine does not modify the probability of progressing from symptomatic to severe disease, because the focus of our analysis is on the primary endpoint of virologically-confirmed disease rather than the more severe outcomes that can sometimes be associated with dengue. Even though dengue vaccines seem to have heterogeneous effects in protection [29], we assumed constant protection with respect to time since inoculation, serotype, and serostatus, to simplify the vaccine model and to isolate the effect of heterogeneous exposure from the effect of heterogeneous vaccine protection.

### Virtual trial procedures

**Fig 1:**
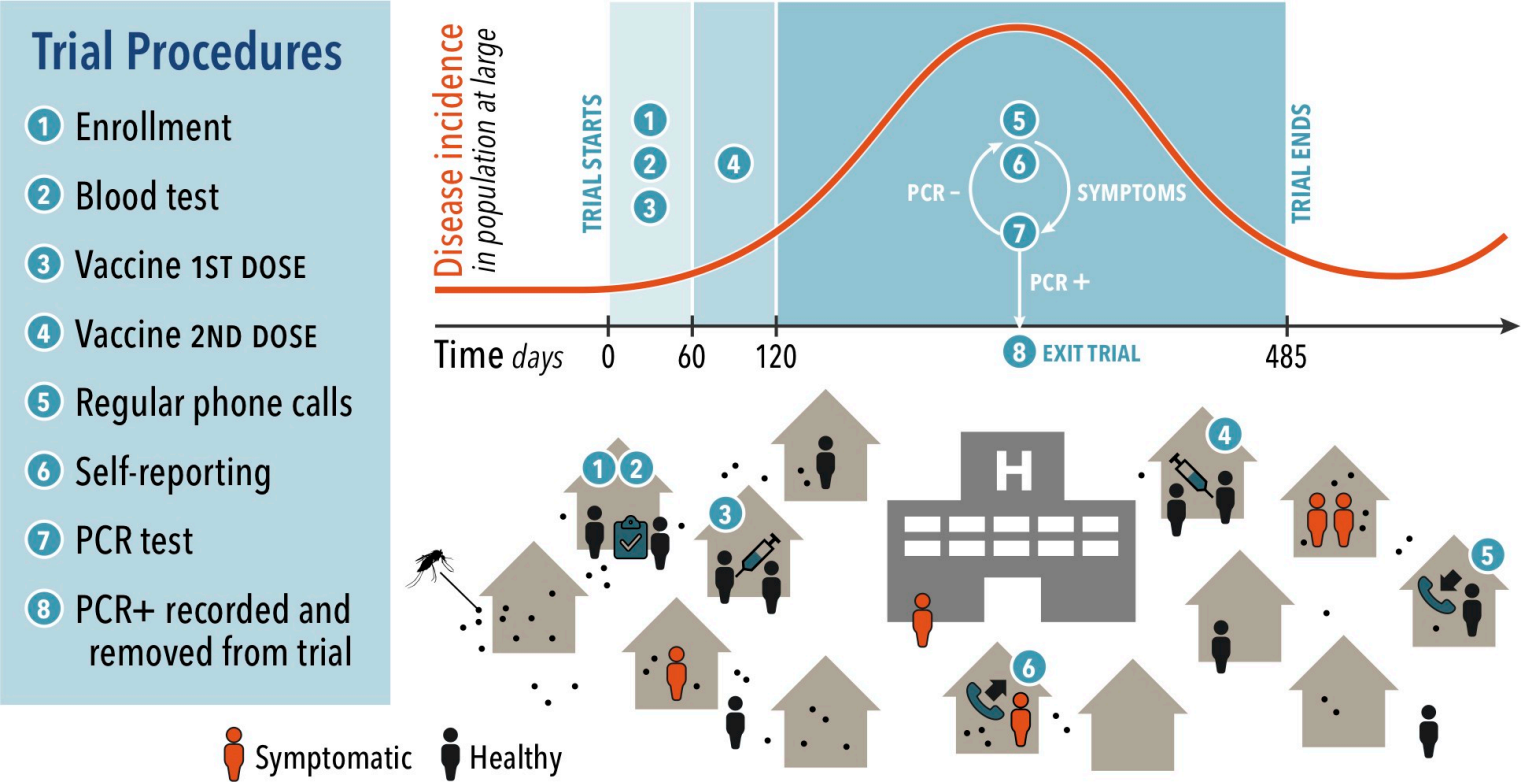
Modeling framework and trial procedures. DENV is transmitted by individual mosquitoes that are found indoors. Humans move daily to different types of locations within the city. Infectious mosquitoes transmit DENV through bites to individuals susceptible to the infecting serotype who are present at a location at the same time as mosquitoes seeking a blood meal. The trial consists of eight procedures listed in the figure. The top curve shows the timing of these procedures in reference to DENV transmission dynamics, and the bottom cartoon depicts each of the procedures.

Our virtual phase-III trial featured three main processes: recruitment, vaccination, and surveillance. These processes comprise eight specific procedures, which are depicted in Fig 1. The recruitment phase included (1) enrollment of participants and (2) serological testing to assess for previous DENV infection. Vaccination included (3,4) administration of two doses of vaccine two months apart. Next, study personnel (5) captured cases during weekly phone calls to participants, and a proportion of (6) individuals with symptoms notified trial personnel. Confirmation of DENV infection (7) was attempted with a serotype-specific RT-PCR test, and (8) confirmed cases were recorded in surveillance reports. The trial considers only the first case of symptomatic dengue for each individual in estimates of VE_S_.

#### Recruitment

We enrolled simulated trial participants during the first two months of the trial. We randomly selected a subset of the simulated population who were 5–45 years old (y.o.). The number of participants was consistent with the desired sample size to achieve a statistical power of 0.9[33], as described in S1 Text. Because DENV exposure differs between children and adults, we recruited participants in such a way that half were children (5–17 y.o.) and half were adults (18–45 y.o.). Within each of those two groups, each participant was randomly assigned to the vaccine or placebo arm on a 1:1 basis. Next, a participant’s pre-existing seropositivity to DENV was recorded, which we assumed could be measured accurately for all trial participants. Individuals without previous exposure to DENV were classified as seronegative and individuals with previous exposure to any of the four DENV serotypes were classified as seropositive, without specification of the number or type of the previous DENV infections.

#### Vaccination

In the simulation, participants received the first dose of the vaccine or placebo on the day of enrollment. The vaccine conferred PEP defined by the vaccine model, whereas the placebo conferred no protection against infection or disease. Each participant received two doses of vaccine or placebo within sixty days of enrollment. This choice was intermediate between two dengue vaccines currently in phase-III trials (Takeda: two doses 90 days apart [32]; Butantan: one dose [31]). We assumed that the full PEP was conferred by the first dose, its longevity boosted by the second dose, and that all trial participants received both doses on schedule. Consistent with observations of modest efficacy despite high immunogenicity in the only dengue vaccine for which efficacy has been assessed [34] and with assumptions of a model developed by a dengue vaccine manufacturer [35], we assumed that the vaccine was leaky: i.e., partial protection applies uniformly to all vaccinees, as opposed to full protection of a subset of vaccinees.

#### Surveillance

Surveillance started on the day on which the first dose of vaccine was administered, but cases used for the VE_S_ analysis were restricted to those that occurred 28 days or more after administration of the second dose, similar to the protocol for the CYD-TDV trials [36]. The surveillance system captured symptomatic cases in two different ways: weekly phone calls to trial participants by trial personnel and self-reporting. Weekly active surveillance contacted participants to inquire about disease symptoms experienced since the last contact with the surveillance system. Half of the individuals with symptoms of dengue fever reported their symptoms during the weekly surveillance phone calls or sought care, which allowed for laboratory diagnosis. Everyone with severe dengue reported their symptoms to the surveillance system. The probability of self-reporting mild dengue cases was higher than estimates of this probability under circumstances outside the context of a trial [37] (5–15%), given that we expect that people would be more likely to use free healthcare provided as part of their participation in the trial. Some DENV infections go undetected because they are asymptomatic or are not virologically-confirmed by the surveillance system. Because these infections are not detected by the surveillance system, their enrollment status in the trial remains unchanged. To confirm the presence of DENV, we simulated an RT-PCR test in participants with two consecutive days of symptoms and up to five days after symptom onset in agreement with current guidelines [38].

To simulate virological confirmation of cases, we modeled the sensitivity of a RT-PCR test as a function of time since symptom onset. To obtain such a function, we first obtained 3,000 simulated viremia trajectories from a model of viremia dynamics for primary and post-primary DENV infections that was fitted to data from individuals enrolled in a clinical study in Vietnam [39]. We then applied a fixed limit of detection (LOD) of 10 cDNA copies per ml to the simulated viremia trajectories and recorded the proportion of those 3,000 simulated trajectories that exceeded that LOD at a given time since symptom onset, which we regarded as the sensitivity of RT-PCR at a given time since symptom onset. We then fitted the function

$$S^{i}\left(t\right)=\;\frac{1}{1+e^{-\left(\beta_{1}^{i}+\beta_{2}^{i}\;t\right)}}$$ (2)

to the simulated values of sensitivity *t* days after symptom onset in primary and post-primary infections *i* using the optim function in R [40], obtaining curves depicted in S1 Fig. We assumed that viremia trajectories corresponding to post-primary infections also applied to vaccinated individuals, given that both should exhibit an antibody response to DENV infection that would result in a shortening of DENV infection and, thus, a shorter window of detectability by RT-PCR.

### Statistical analyses

We used surveillance records resulting from a given virtual vaccine trial to estimate VE_S_ based on the timing of when trial participants attained the primary endpoint as detected by the surveillance system. The model simulated censored observations; i.e., some individuals dropped out of the trial before being infected, whereas others finished the trial without being exposed to DENV. Ignoring this feature of the data can result in overestimation of vaccine efficacy [19]. We estimated the vaccine efficacy for susceptibility (VE_S_) as a measure of how protective the vaccine was against disease [19]. VE_S_ was calculated based on three approaches: incidence rates, survival methods, and transmission probability. Based on incidence rates, VE_S_,_IR_ was calculated as

$${\text{VE}}_{\text{S},\text{IR}}=1-\frac{c_{v}/y_{v}}{c_{p}/y_{p}},$$ (3)

where *c* represents the number of cases detected in each arm of the study, *y* the number of person-years within the trial, and the subscripts *v* and *p* represent the vaccine and placebo arms, respectively [19]. To calculate VE_S_ based on survival methods, we estimated VE_S,Cox_ with a Cox model in R [40] using the coxph function with default settings [41]. The survival model was implemented with the data represented by the time to first event (or follow-up time) and a binary variable for PCR positivity. In an attempt to account for heterogeneous exposure within the trial, we also considered the addition of a frailty expression—in which heterogeneity in exposure was defined as a random effect by a gamma distribution—to the Cox-regression model and estimated VE_S,frailty_ using the same coxph function in R with an additional frailty term specified as frailty(ID, distribution = "gamma").

The above methods do not require information about the level of exposure of individuals in the trial. Such measures are often used in phase-III trials to measure VE_S_ because information on exposure to infection may be unfeasible to obtain in field trials. This task is particularly difficult for mosquito-borne diseases, because individual mosquito bites would need to be recorded to estimate the level of exposure of trial participants. Our model allowed us to record the exact number of exposures that every individual experienced during the trial, defined as an infectious bite received by a susceptible human. Knowledge on the exact number of exposures allowed us to estimate VE_S_ based on the probability of transmission ‘p’, which should be robust to heterogeneous exposure. The measure VE_S,p_ was defined as

$${\text{VE}}_{\text{S},\text{p}}=1-\frac{c_{v}/n_{v}}{c_{p}/n_{p}},$$ (4)

where *c*_*v*_ and *c*_*p*_ represent the number of cases in the vaccine and placebo arm, respectively, and *n*_*v*_ and *n*_*p*_ represent the total number of exposures in the vaccine and placebo arms, respectively [19]. Comparison between this and other VE_S_ measures was used to aid in the interpretation of our results.

### Model overview

We simulated vaccine trials using an agent-based model of DENV transmission that simulates the transmission dynamics of DENV in a population of 200,000 in a core population area of Iquitos, Peru [42,43]. Our model includes simulated daily human movement patterns calibrated to data from retrospective, semi-structured interviews of residents in Iquitos [44,45]. This model has been demonstrated to reproduce the dynamics of all four DENV serotypes [42] and was used to investigate the epidemiological impacts of hypothetical vaccination campaigns to project the population-level impact of Dengvaxia [46,47]. Because a formal description of the version of the transmission model that was used here has been published elsewhere [42], we provide an overview of its key features and assumptions in S2 Text.

### Model calibration

We used parameter estimates from the literature wherever possible (Table 2) and calibrated the values of unknown parameters so as to allow the model to generate patterns of transmission within the study area consistent with time-varying incidence of infection estimates from a longitudinal cohort study [27]. Three sets of parameters were calibrated through this process: (1) serotype-specific population immunity on 1 January 2000, when estimates of time-varying, serotype-specific incidence of infection began; (2) time-varying, serotype-specific patterns of unexplained infections from visitors to the study area or residents that were infected outside the study area; and (3) constants for scaling mosquito population density at two different time points during which mosquitoes were collected using different methods. We calibrated these parameters using a particle filtering algorithm described in detail elsewhere (Table 3) [42].

**Table 2:** Fixed parameters of the model.

Parameter	Value	Reference
Mosquito daily movement probability to a nearby house	30%	[48]
Mosquito daily emergence rates	Consistent with time-varying estimates of adult mosquito death rates and spatiotemporal estimates of Ae. Aegypti density in Iquitos.	[49,50]
Average interval between blood feeding events (exponential distribution)	Temperature-dependent	[51–53]
Probability of pathogen transmission to susceptible human conditional on an infectious mosquito bite	1.0	[54]
Incubation period in the mosquito (lognormal distribution)	μ=f(T) , σ=0.451754	[55]
Infectiousness—human symptomatic primary infection	$e^{\left(-0.204{\left(t-t_{i}-5.538\right)}^{2}/0.999\right)}$	[56]
Infectiousness—human symptomatic secondary infection	$e^{\left(-0.383{\left(t-t_{i}-5.874\right)}^{2}/0.999\right)}$	[56]
Infectiousness—human asymptomatic primary infection	$e^{\left(-0.25{\left(t-t_{i}-5.585\right)}^{2}/0.999\right)}$	[56]
Infectiousness—human asymptomatic secondary infection	$e^{\left(-0.584{\left(t-t_{i}-4.883\right)}^{2}/0.999\right)}$	[56]
Incubation period in humans	Captured by time-varying infectiousness curve	[55,56]
Human temporary immunity period (Exponential distribution).	$\lambda =\frac{1}{686}\;days^{-1}$	[42,57]
Probability of developing symptoms given primary infection	30%	[56]
Probability of developing symptoms for secondary infection	60%	[56]
Probability of developing symptoms for tertiary and quaternary infections	10%	[56]
Probability of severe disease for primary infection, given the apparition of symptoms	11%	[56]
Probability of severe disease for secondary infection, given the apparition of symptoms	20%	[56]
Probability of severe disease for tertiary and quaternary infections, given the apparition of symptoms	5%	[56]
Human demographic rates	Consistent with U.N. estimates	[58]

**Table 3:** Calibrated parameters.

Parameter	Reference
Serotype-specific population immunity on 1 Jan. 2000	Fitted to represent estimates of force of infection [27]
Time-varying, serotype-specific patterns of imported infections	Fitted to represent estimates of force of infection [27]
Scaling of mosquito population density	Fitted to represent estimates of force of infection [27]

### Simulation experiments

Our primary goal was to quantify bias in VE_S_ estimates insofar as they relate to estimates of individual protection afforded by the vaccine, as defined in our simulation model by PEP. This individual-level interpretation is significant, because VE_S_ estimates from standard methods represent a weighted average of vaccine effects at the population level. Therefore, whenever the assumption of homogeneous exposure is not met, VE_S_ estimates are specific to exposure patterns in the population where the vaccine was evaluated [22]. To the extent that VE_S_ estimates made in this way might be used to inform projections of vaccination impact in other populations (due to what is usually a lack of a direct estimate of PEP), understanding the extent of bias in estimates of PEP based on VE_S_ is important [22]. To address this driving question, we performed three simulation experiments under different assumptions about heterogeneity in DENV transmission patterns.

#### 1. Baseline scenario

The trial design under the baseline scenario was described in the Trial Overview and Sample Size sections. In this scenario, the attractiveness of humans to mosquitoes was proportional to the human’s body size [11]. The timeframe of the trial ranged one to three years depending on when 68 virologically-confirmed symptomatic dengue cases were captured. We simulated 1,000 replicates of this trial with an input PEP = 0.8 and quantified the bias of the estimated VE_S_ relative to the input PEP. We also simulated the trial with an alternative assumption about the viremia of infected vaccinees, in which their viremia followed that of someone experiencing a natural DENV infection. We quantified differences between estimated VE_S_ and simulated PEP under both assumptions of viremia of infected vaccinees.

We computed the probability that the point estimate of VE_S_ was lower than a specified minimum product profile (MPP) below which the vaccine would not be licensed or further developed. We varied the value of MPP (0.1–0.7) and calculated this probability given a PEP equal to the target product profile (TPP) (VE ≥ 0.8). We varied the value of PEP (0.1–0.95) and computed the proportion of trials resulting in VE_S_ falling below MPP = 0.3.

#### 2. Effects of heterogeneous exposure due to heterogeneous biting

**Fig 2:**
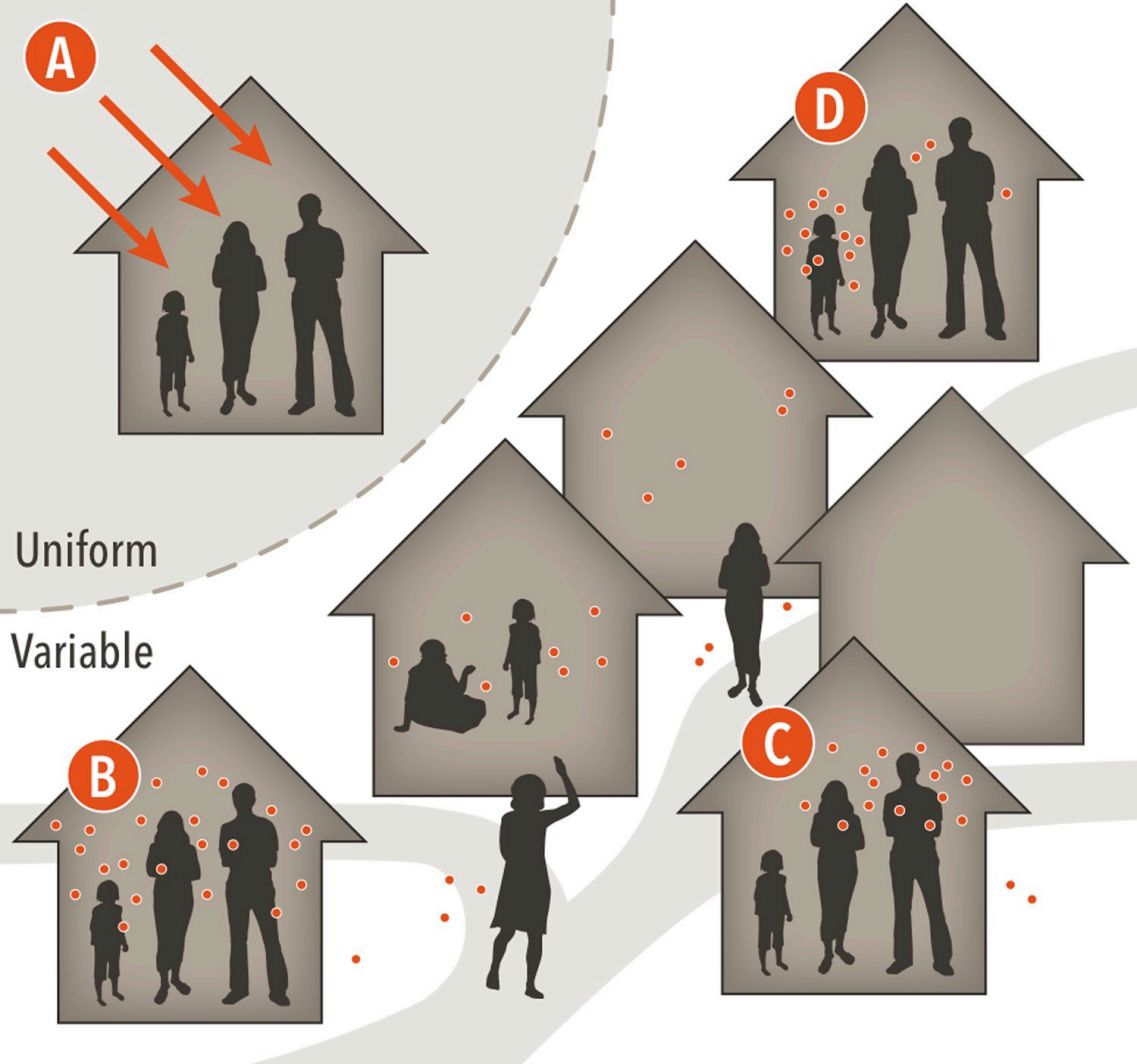
Four distinct assumptions about heterogeneous DENV exposure. Scenario A represents homogeneous transmission of DENV ignoring mosquitoes and human movement patterns, leading to everyone experiencing the same hazard of exposure to any serotype of DENV. In scenarios B, C, and D, DENV is transmitted by mosquitoes, and human and mosquito movement patterns are considered. Scenario B represents homogeneous attractiveness of different humans to mosquitoes at a given location. Scenario C represents human attractiveness to mosquitoes proportional to human body size. Scenario D represents heterogeneous human attractiveness to mosquitoes based on a gamma distribution with shape 1.43 and scale 1, regardless of their body size.

Heterogeneity in the number of DENV exposures experienced by participants in each trial arm can affect trial results. In our model, an exposure is defined as a bite from an infected mosquito that would result in a human infection in the absence of vaccination. Hence, placebo participants can only experience one exposure before developing infection. Conversely, participants in the vaccine arm can experience multiple exposures before infection due to the assumption that the vaccine is leaky. We simulated four scenarios about the nature of heterogeneous exposure, illustrated in Fig 2, to investigate how different forms of exposure heterogeneity affect VE_S_ estimates.

The different forms of exposure heterogeneity that we considered (Fig 2) come about through:

- Homogeneous hazard of exposure, in which mosquitoes are bypassed altogether and individuals are infected directly based on a time-varying rate of DENV infection.
- Constant attractiveness, in which mosquitoes are equally attracted to all humans for blood feeding at a location.
- Attractiveness proportional to body surface area, in which mosquitoes are more likely to bite humans with larger bodies [11].
- Heterogeneous attractiveness, in which each individual’s attractiveness is drawn from a gamma distribution with shape α = 1.43 and rate β = 1, as informed by data from de Benedictis et al. [12].

In addition to heterogeneity in attractiveness to mosquitoes, heterogeneous exposure in the transmission model results from spatial heterogeneity in contact between mosquitoes and humans, due to spatial patterns of mosquito abundance and human mobility. This is true for all scenarios except A. In scenario A, infections during a trial occur only by a time-varying hazard of DENV infection. We adjusted this hazard to be the same as the force of infection in the remaining three scenarios so that within the trial the AR would be comparable among scenarios. We compared the distributions of VE_S_ estimates under these four scenarios by performing 1,000 simulations, with analogous simulations in each set that had the same sequence of random number seeds to minimize differences due to chance across the four scenarios.

#### 3. Effect of increased heterogeneity of exposure due to high transmission intensity

We hypothesized that phase-III trials in sites with high transmission intensity yield more biased vaccine efficacy estimates than trials performed with moderate to low transmission intensity, due to an increased number of exposures in the vaccine arm. Our goal was to quantify the potential magnitude of this effect in our simulated trials by computing bias in VE_S_ estimates in a scenario with high transmission intensity. We achieved variation in transmission intensity by varying mosquito emergence rates, which scale the overall density of adult mosquitoes and have a linear effect on the force of infection. Specifically, we doubled mosquito emergence rates in the high-transmission scenario, and we calculated required sample sizes separately for each trial to attain the same statistical power for VE_S_ estimates under both scenarios. With these specifications, we simulated 1,000 replicates for each scenario with paired random seeds to allow the behavior of the simulations to be as comparable as possible.

## Results

### 1. Baseline scenario

**Fig 3:**
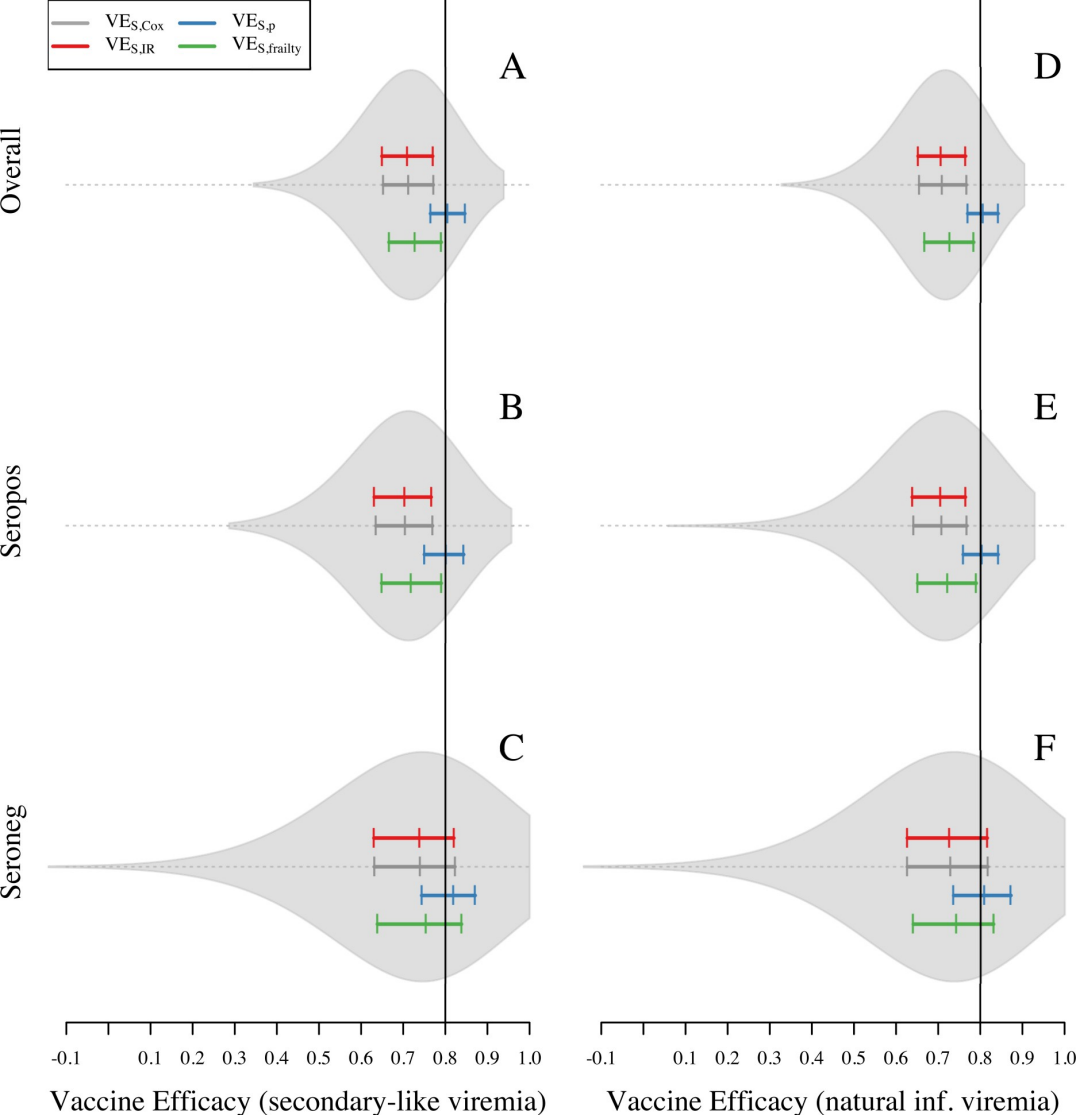
Overall VE_S_ and VE_S_ with stratification estimated for 1,000 simulations of the baseline scenario for one site. In this scenario, attractiveness of humans is proportional to their body size. VE_S_ is calculated with four different methods: VE_S,Cox_, VE_S,IR_, VE_S,CI_, and VE_S,p_. Violin plots on the left panel show the shape of the distribution of VE_S,Cox_ estimates. Colored solid lines show the 25, 50, and 75 quantiles for VE_S,Cox_, VE_S,p_, VE_S,IR_, and VE_S,frailty_ (gray, blue, red, and green, respectively). Black solid line represents the true PEP = 1—RR_inf|exp_ RR_dis|inf_ = 0.8. Left panel corresponds to the assumption of viremia in vaccinated individuals as secondary infections. Right panel corresponds to the assumption of viremia in vaccinated individuals as a natural infection.

Analysis of the baseline simulations showed a general pattern of VE_S_ underestimating the simulated value of PEP (0.8) when using VE_S,IR_ (0.71) and VE_S,Cox_ (0.71). Compared to VE_S,IR_ and VE_S,Cox_, the frailty model slightly reduced bias in its estimate of VE_S,frailty_ (0.73). Knowledge of the number of exposures in each trial arm was associated with a slight overestimation of efficacy by VE_S,p_ (0.81) (Fig 3A). Although these patterns held qualitatively across the full range of assumptions about the extent to which the vaccine protects against infection versus disease (modulated by the parameter ρ), the magnitude of bias in VE_S,IR_, VE_S,Cox_, and VE_S,frailty_ was somewhat sensitive to ρ (S3 Fig). Specifically, these biases were stronger when protection derived more from reducing the risk of infection conditional on exposure and less from reducing the risk of disease conditional on infection (i.e., ρ closer to 1). The slight upward bias of VE_S,p_ by 0.01 was unexpected, because VE_S,p_ should be capable of fully accounting for bias due to heterogeneous exposure [19]. We also noted that the extent of bias was different between seropositive (Fig 3B) and seronegative (Fig 3C) groups. In particular, bias towards the null was 0.02 higher in the seropositive group for all VE_S_ measures (Fig 3B). Even though this was a small difference, an upward bias in the seronegative group could partially mask the magnitude of the bias due to heterogeneous exposure in the overall VE_S_ estimates.

After reviewing aspects of the simulation model that could have differed between seropositive and seronegative individuals, we hypothesized that a bias away from the null that is distinct from the bias due to heterogeneous exposure occurred as a result of the way in which we simulated viremia trajectories in infections of vaccinated individuals. Because these infections were defined as secondary-like infections with shorter viremia periods (S1 Fig), fewer primary infections in the vaccine arm were detected by the RT-PCR than in the placebo arm. This resulted in VE_S_ estimates being approximately 0.01 higher on average than they would have been if the viremia trajectories in primary vaccinee infections had been the same as they were in unvaccinated individuals (Fig 3C and 3F). This bias increased to 0.06 (S2C and S2F Fig) in a scenario in which RT-PCR was used up to seven days after symptom onset, in contrast to the baseline of five days. This effect was apparent for seronegative individuals (for whom there were differences in RT-PCR sensitivity between vaccinated and unvaccinated) but not for seropositive individuals (for whom RT-PCR sensitivity was equal for vaccinated and unvaccinated) (Fig 3B and 3C). We interpret this bias to have occurred because differences in RT-PCR sensitivity among seronegative trial participants resulted in vaccinated individuals being perceived to experience an artificially low number of infections. As expected under this interpretation, this bias disappeared in simulations in which we assumed that RT-PCR sensitivity was the same for vaccinated and unvaccinated individuals (Figs 3F and S2F). Furthermore, these individuals were also protected from infection later in the trial, which was perceived to result from vaccination but in reality resulted from heterotypic immunity following natural infection. Assumptions about viremia did not affect VE_S_ estimates for the seropositive population (Fig 3B and 3E), because their viremia curves were the same for vaccinated and unvaccinated individuals.

Because differential detectability resulted in a bias away from the null under the baseline scenario, it had the effect of slightly reducing our estimate of the extent to which heterogeneous exposure induced bias toward the null. VE_S_ estimates from the seropositive group were unaffected by the bias due to differential detectability and, therefore, provided a clearer indication of the extent of bias due to heterogeneous exposure. Across all four measures of VE_S_ that we examined, bias was approximately 0.01 more toward the null in the seropositive group than in the trial as a whole (Fig 3F vs 3A). This relatively small difference between the overall VE_S_ estimate and the VE_S_ estimate for the seropositive group can be attributed to the majority of trial participants being seropositive (0.6, CI = [0.57,0.63]) and the majority of the cases coming from seropositive individuals (0.66, CI = [0.55, 0.76]). Consequently, the extent to which bias away from the null due to differential detectability counteracts bias toward the null due to heterogeneous exposure would be expected to differ in trials in which the proportion of seropositive trial participants differs from our simulations. In summary, using conventional measures of VE_S_ that do not account for these biases (i.e., VE_S,Cox_ and VE_S,IR_), analyses of our simulations with PEP = 0.8 resulted in VE_S_ estimates that were biased toward the null by approximately 0.09 due to heterogeneous exposure. Estimates of VE_S_ were biased away from the null by as much as 0.01 due to differential detectability of primary infections between vaccinated and unvaccinated individuals.

**Fig 4:**
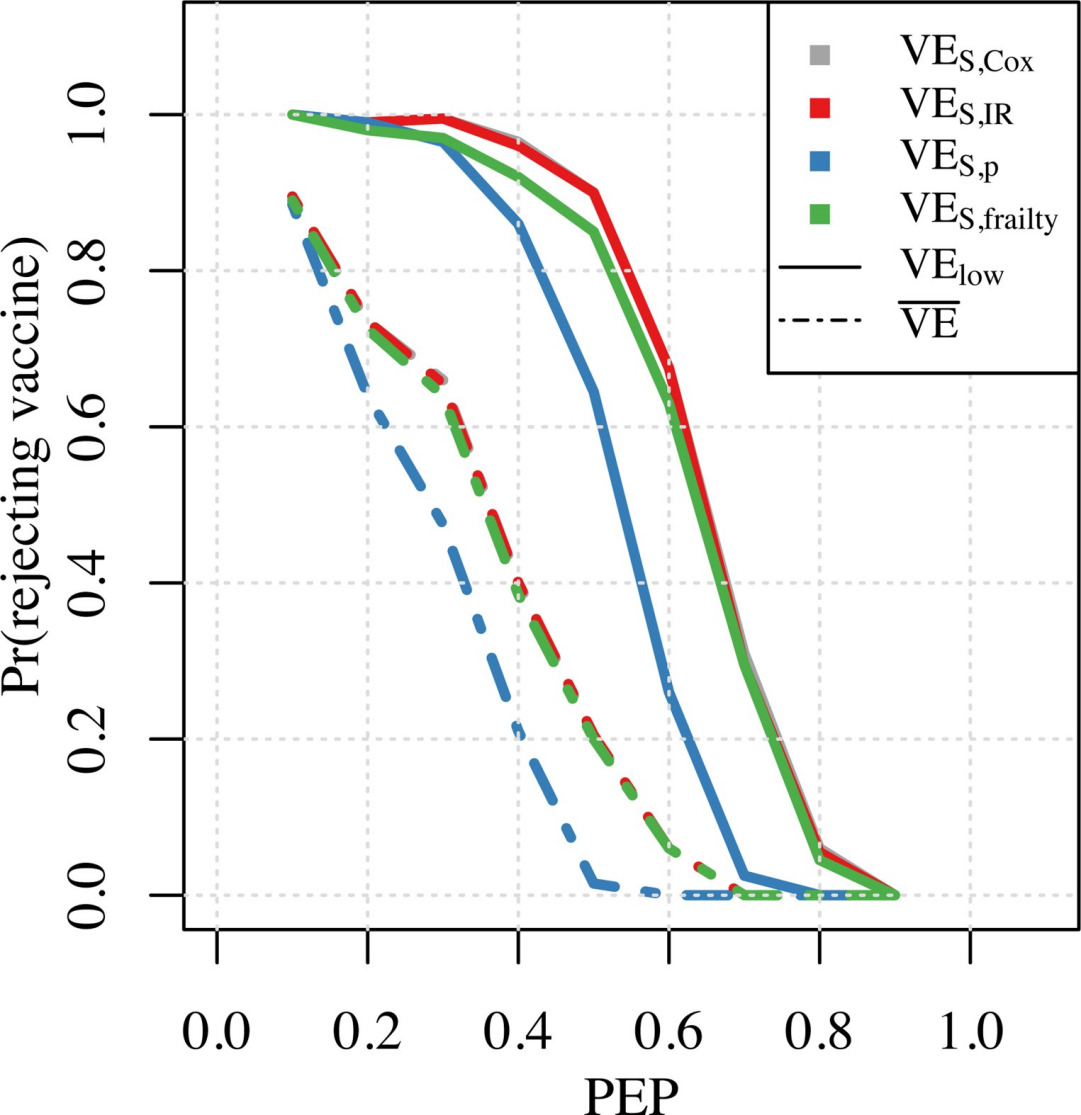
Probability of not licensing a vaccine with MPP = 0.3 at different input PEP values. Solid lines represent the probability of rejection calculated as the proportion of simulations where the lower bound of the confidence interval of VE_S_ was below MPP. Dotted lines represent the probability of rejection calculated as the proportion of simulations where the mean estimate of VE_S_ was below MPP.

As one way to place these biases into context, we examined the proportion of simulated trials in which the vaccine would not have been licensed on the basis of the VE_S_ point estimate falling below the minimum product profile (MPP). For an MPP of 0.3, this probability depended on the VE_S_ measure used at different values of PEP. This probability was always lower for the VE_S,p_ measure, compared to the other three measures used (Fig 4). For instance, for PEP = 0.4, the vaccine would not have been licensed in nearly 40% of simulated trials on the basis of the point estimates of VE_S,Cox_, VE_S,frailty_, and VE_S,IR_. It would not have been licensed in almost all simulated trials if the lower bound of the confidence interval of the VE_S_ estimates were used as a criterion for licensure. For PEP = 0.4, the probability of a vaccine not being licensed on the basis of the point estimate of VE_S,p_ was only around 0.2, and around 0.8 when based on the lower bound of the confidence interval. Across these and other instances, there was a lower probability of rejecting a vaccine with PEP exceeding MPP whenever PEP was farther away from MEP and when bias toward the null was less severe.

### 2. Effects of heterogeneous exposure due to heterogeneous biting

**Fig 5:**
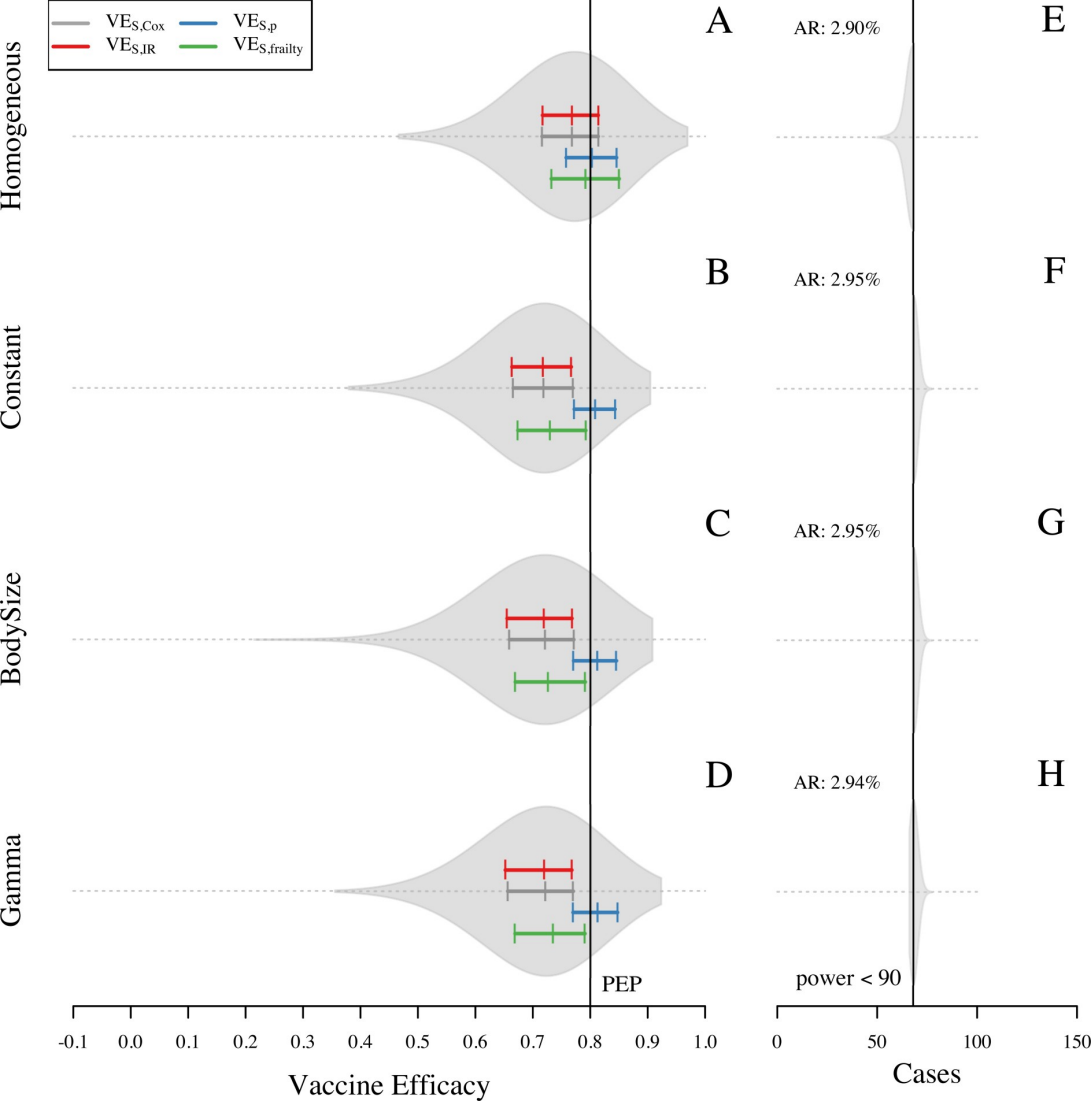
Vaccine efficacy in four scenarios about heterogeneous exposure to DENV for seropositive and seronegative individuals. Violin plots show the shape of the distribution of VE_S,Cox_ estimates. The colored solid lines show the 25, 50, and 75 quantiles for VE_S,Cox_, VE_S,p_, VE_S,IR_, and VE_S,frailty_ (gray, blue, red, and green, respectively). Black solid line represents the individual input of vaccine efficacy: VE = 1 –PEP = 1—RR_inf|exp_ RR_dis|inf_ = 0.8. The left panel corresponds to VE_S_ in seropositives and the right panel corresponds to the VE_S_ in seronegatives.

**Fig 6:**
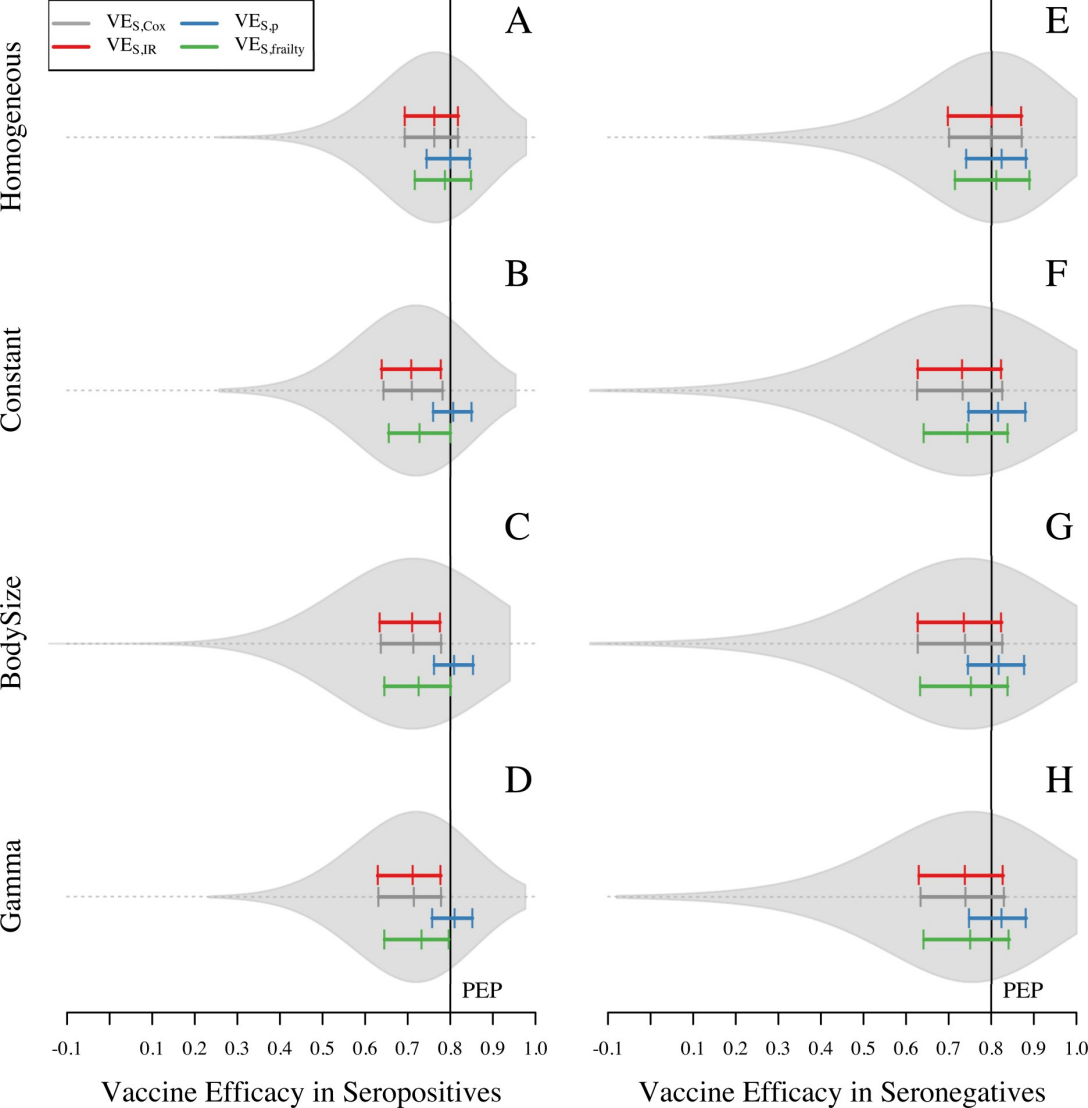
Vaccine efficacy in four scenarios about heterogeneous exposure to DENV for seropositive and seronegative individuals. Violin plots show the shape of the distribution of VE_S,Cox_ estimates. The colored solid lines show the 25, 50, and 75 quantiles for VE_S,Cox_, VE_S,p_, VE_S,IR_, and VE_S,frailty_ (gray, blue, red, and green, respectively). Black solid line represents the individual input of vaccine efficacy: VE = 1 –PEP = 1—RR_inf|exp_ RR_dis|inf_ = 0.8. The left panel corresponds to VE_S_ in seropositives and the right panel corresponds to the VE_S_ in seronegatives.

The scenario with homogeneous hazard of exposure was associated with less bias toward the null compared to the three scenarios that allowed for some form of heterogeneous hazard of exposure (Fig 5A compared with Fig 5B–5D). When the hazard of exposure was homogeneous, VE_S,p_ and VE_S,frailty_ were approximately equal to PEP (VE_S,p_ = 0.8, VE_S,frailty_ = 0.79), and the other two measures slightly underestimated it (VE_S,Cox_ = 0.77, VE_S,IR_ = 0.77) (Fig 5A). As in the baseline scenario described above, overall estimates of VE_S_ reflect a combination of distinct VE_S_ estimates for the seropositive and seronegative groups, due to a bias away from the null resulting from differential detectability of primary infections among vaccinated and unvaccinated individuals. Among seropositive individuals, VE_S,p_ was the only measure that appeared to be unbiased (blue in Fig 6A). The other three measures exhibited some bias toward the null (red, gray, green in Fig 6A), because a homogeneous hazard of exposure still results in some degree of heterogeneity in the numbers of exposures that individuals experience (S4 Fig). Among seronegative individuals in the vaccinated group, the downward bias of heterogeneous exposure canceled out with the upward bias of lower detectability of primary infections and VE_S,Cox_ and VE_S_,_IR_ showed unbiased estimates of VE_S_, while VE_S,p_ and VE_S,frailty_ displayed bias away from the null for this group (Fig 6E).

The scenarios with heterogeneous hazard of exposure resulted in a larger bias toward the null when using VE_S,Cox_, VE_S,IR_, and VE_S,frailty_ than when using VE_S,p_ (blue closer to PEP than gray, red, or green in Fig 5B–5D). These estimates were all within approximately 0.01 of each other under all three scenarios that allowed for heterogeneous attractiveness of humans to mosquitoes (right three columns in S5 Fig), as opposed to a difference of approximately 0.05 between the homogeneous scenario and each of the three heterogeneous scenarios (left three columns in S5 Fig). This indicates that a substantial degree of heterogeneity in exposure derives from spatial heterogeneity in mosquito density among different houses and heterogeneity in how individual humans sample that heterogeneity through their movement patterns, whereas relatively little derives from heterogeneous blood feeding within a single house. Examination of the distribution of the number of exposures experienced by trial participants further supports this conclusion, with higher variance in the number of exposures under the three heterogeneous scenarios than under the homogeneous scenario (S4 Fig).

### 3. Effect of increased heterogeneity of exposure due to high transmission intensity

**Fig 7:**
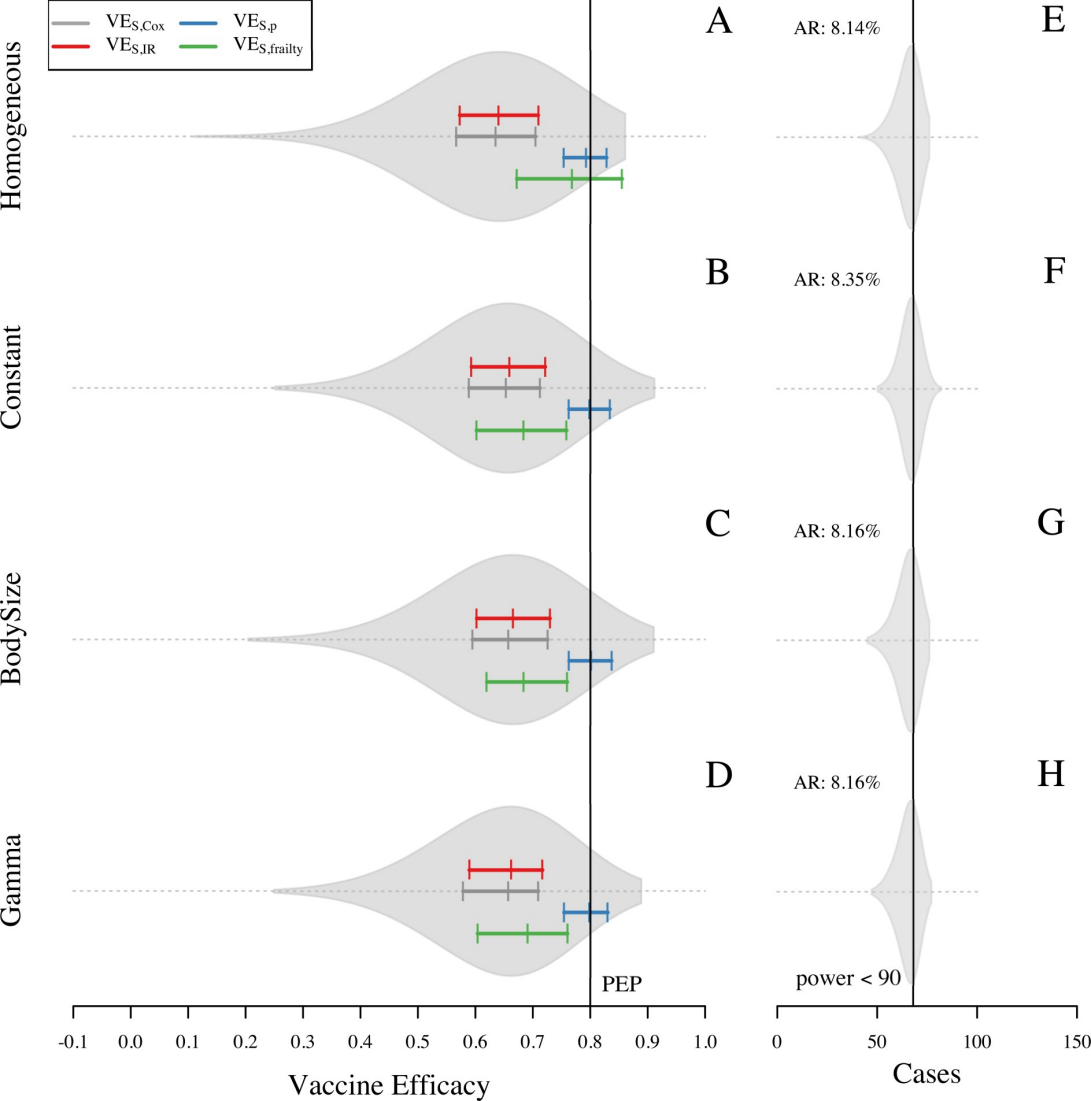
Overall VE_S_ estimates from 1,000 simulations of each setting with different assumptions about heterogeneous exposure in a high-intensity setting. Sample size was adjusted to 800 participants to achieve 90% statistical power in a higher transmission setting. Each row represents an assumption about heterogeneous exposure to DENV: homogeneous hazard of exposure, constant attractiveness of humans, attractiveness of humans based on body size, and attractiveness of humans based on a gamma distribution (shape = 1.43 and scale = 1). VE_S_ is calculated with four different methods: VE_S,Cox_, VE_S,IR_, VE_S,CI_, and VE_S,p_. Violin plots on the left panel show the shape of the distribution of VE_S,Cox_ estimates. Colored solid lines show the 25, 50, and 75 quantiles for VE_S,Cox_, VE_S,p_, VE_S,IR_, and VE_S,frailty_ (gray, blue, red, and green, respectively). Black solid line represents the true PEP = 1—RR_inf|exp_ RR_dis|inf_ = 0.8. Right panel corresponds to the number of cases captured in the trial.

Higher transmission intensity increased the bias in VE_S_ estimates even further toward the null because of a higher chance of repeated exposure in the population at risk, which increased heterogeneity in the number of exposures. This increased heterogeneity also affected the scenario with homogeneous hazard of infection (Fig 7A), because homogeneity in the hazard of exposure does not ensure homogeneity in the number of exposures that individuals experience during the trial. Some individuals in this scenario were exposed up to five times during the trial (S6 Fig), compared to a maximum of three exposures under the baseline transmission intensity assumptions (S4 Fig). Under the homogeneous hazard scenario, the frailty model did a relatively good job of reducing bias toward the null (VE_S,frailty_ = 0.77, compared with VE_S,Cox_ = 0.64, VE_S,IR_ = 0.64), but it was less successful at doing so under the three heterogeneous hazard scenarios (VE_S,frailty_ = 0.69, compared with VE_S,Cox_ = 0.66, VE_S,IR_ = 0.66) (Fig 7B–7D). Accounting for exposure with VE_S,p_ resulted in unbiased estimates.

## Discussion

Our results indicate that, under the assumption of a leaky vaccine (i.e., provides partial protection to all vaccinated individuals), VE_S_ is consistently underestimated by standard measures that do not account for the repeated, heterogeneous nature of DENV exposure. For instance, heterogeneity in exposure violates an assumption required by the Cox model to ensure unbiased estimates of VE_S_ [22]. Heterogeneous exposure has the effect of reducing efficacy because some individuals are highly protected from infection in both arms of the study, which makes the placebo arm of the study more similar to the vaccine arm. Under the default assumptions and scenarios that we examined, we observed that the extent of bias toward the null as a result of heterogeneous exposure could be as great as 21% of the value of simulated PEP, depending on the extent to which the vaccine protects against infection or disease. Depending on the simulated value of PEP and how close it was to MPP, we found that different methods for estimating VE_S_ have implications for the possibility of not licensing a vaccine that has desirable properties but is not perceived as such due to bias from heterogeneous exposure.

One possibility for mitigating bias due to heterogeneous exposure that we examined was incorporating a frailty expression, which accounts for unmeasured heterogeneity as a random effect [21], into our Cox survival analysis of simulated trial data. This step did help reduce VE_S_ bias, particularly under scenarios in which heterogeneity was more extreme. Although our results indicate that incorporation of a frailty term into estimation of VE_S_ from dengue vaccine trial data could help reduce bias toward the null, a caveat is that biased estimates of VE_S_ can still be obtained when the frailty distribution is not accurately specified [21]. Also, at high degrees of censoring, non-identifiability of the model parameters can be an issue with frailty models [21].

Another possibility for mitigating bias due to heterogeneous exposure is to incorporate information about the number of exposures in each trial arm. This approach successfully eliminated bias due to heterogeneous exposure in our simulations. A disadvantage, however, is that additional information pertinent to the number of exposures in each trial arm must be collected. In a real trial, doing so would amount to obtaining estimates of the total number of bites by infectious mosquitoes on all individuals in each trial arm. Even as an average across many individuals in a trial arm, estimating entomological risk of DENV infection is not straightforward [59]. Challenges to doing so include significant spatiotemporal heterogeneity in DENV transmission activity [7], shifting hotspots of mosquito density over timescales shorter than a trial [15], and the fact that individuals are subject to mosquito bites and DENV exposure at the numerous locations they visit during their daily routines [60]. Unfortunately, these are precisely the forms of heterogeneity that our results indicate contribute most to bias in VE_S_ estimates, rendering VE_S,p_ an ideal, but impractical, solution to mitigate bias due to heterogeneous exposure.

We also found an unexpected source of bias for seronegative individuals. This bias comes from our assumption of shorter viremia periods in primary infections for vaccinated compared to unvaccinated individuals. This assumption resulted in fewer detected cases between follow-up visits in the vaccine arm than in the placebo arm. As a consequence, estimates of VE_S_ in the seronegative group were biased away from the null. This bias was relatively small when the RT-PCR was used up to five days after symptom onset, as advised by the Centers for Disease Control and Prevention [38]. Not adhering to these guidelines could, however, result in larger bias away from the null, which we observed when we allowed use of RT-PCR up to seven days after symptom onset. Ignoring this type of bias could result in reducing the magnitude of the bias toward the null from heterogeneous exposure. Doing so may be acceptable from the perspective of estimating overall VE_S_, but it has the potential to bias estimates of VE_S_ for seronegatives above that of seropositives. For biological reasons [61], estimates for Dengvaxia indicate that VE_S_ is higher for seropositives than seronegatives [29]. If analyses of Dengvaxia trial data are subject to this bias from differential detectability, that would indicate that the estimated difference in VE_S_ between seropositives and seronegatives could be somewhat greater than currently thought.

There are also sources of bias other than heterogeneous exposure and differential detectability that could manifest in dengue vaccine trials. For instance, infections among trial participants that go undetected could result in bias toward the null. This could occur if analyses of symptomatic endpoints fail to consider that unobserved infections in the placebo arm differentially reduce person-time at risk, increasing the apparent similarity between outcomes in vaccine and placebo arms [62]. This effect may have been moderated in our analyses because conditions that promote it—protection deriving from blocking infection rather than ameliorating disease, high baseline hazard—were not in effect to a large degree. Nonetheless, undetected DENV infections are common [63] and can cause other problems for the interpretation of dengue vaccine trial data [64]. Heterogeneity in susceptibility is a related source of bias that causes bias toward the null for reasons similar to why unobserved infections do [65]. Although pre-enrollment serological testing could be used to classify individuals as seropositive or seronegative for any dengue vaccine trial, it is likely that the serotype(s) to which an individual is susceptible would be undefined, which could be considered a potential source of unmeasured heterogeneity in susceptibility.

Recent decades have seen great elaboration of theory for vaccine trial design and analysis [19], which has increasingly embraced heterogeneity in data-generating processes such as exposure. This growing body of literature has resulted in a number of principles about potential sources of bias in VE_S_ estimation, highlighted through simulation experiments that have tended to make use of relatively simple models [66]. Because our primary goal in this study was to quantify the extent of a known bias for a specific disease, we opted for an agent-based model that uses highly detailed data to realistically simulate effects of heterogeneous exposure. In addition to achieving this explicit goal of our study, we uncovered an unexpected source of bias that could have implications for interpretation of Dengvaxia trial data. This discovery emerged from our analyses as a result of including detail in model structure beyond what was minimally necessary to meet our primary goal of quantifying bias due to heterogeneous exposure. This work adds to a growing list of applications of dynamic transmission models to planning for dengue vaccine trials (this study), interpreting their results [35,61], and making projections of their impacts when deployed at the population level [42,46,47,61].

## Supporting information

S1 Text Statistical estimation of sample size. (DOCX)

S2 Text Agent-based model of dengue virus transmission. (DOCX)

S1 FigSensitivity as a function of time since symptom onset for primary and post-primary DENV-infected individuals given a limit of detection of 10 cDNA copies/mL.Dots represent the sensitivity curves obtained from 3,000 simulations of viremia. Solid lines represent the fitted curves described in Eq 1. For primary infections, $\beta_{1}^{1}\;=\;13.18506$ and $\beta_{2}^{1}\;=\;-1.665468$. For post-primary infections, $\beta_{1}^{2}\;=\;6.834631$ and $\beta_{2}^{2}\;=\;-1.166282$.(PDF)

S2 Fig Overall VE_S_ and VE_S_ with stratification estimated for 1,000 simulations of the baseline scenario for one site with RT-PCR up to 7 days after symptom onset. Attractiveness of humans is proportional to their body size. VE_S_ is calculated with four different methods: VE_S,Cox_, VE_S,p_, VE_S,IR_, and VE_S,frailty_ (gray, blue, red, and green, respectively). Left panel shows results under the assumption that viremia in vaccinated individuals is similar to that in secondary infections. In the right panel, viremia in vaccinees is the same as a natural infection. Violin plots show the shape of the distribution of VE_S,Cox_ estimates. Colored solid lines show the 25, 50, and 75 quantiles for VE_S_. Black solid line represents the individual input of PEP = 1—RR_inf|exp_ RR_dis|inf_ = 0.8.
(EPS)

S3 Fig Sensitivity of overall VE_S_ to changes in the proportion of protection from infection. Each panel shows VE_S_ estimates across different values of the parameter p. Dots show the estimates for each of the four VE_S_ measures: VE_S,Cox_, VE_S,p_, VE_S,IR_, and VE_S,frailty_. The black line shows the regression obtained with a generalized additive model and the dark-gray band shows the 95% confidence interval. In all scenarios, PEP is represented by the black dotted line and is defined as PEP = 1 –RR_inf|exp_ RR_dis|inf_ = 0.8. The proportion of protection from infection, p, is defined such that RR_inf_ = (1 –PEP)^ρ^_,_ and RR_dis|inf_ = (1 –PEP)^1 –ρ^_._
(EPS)

S4 Fig Distribution of exposures within the vaccine group of the trial for each of four assumptions of heterogeneity of exposure. The height of the bars represents the relative frequency of the number of exposures in the vaccine arm for a specific realization of the virtual trial. The four simulations used the same random number seed to increase comparability among simulations.
(EPS)

S5 Fig Pairwise difference of VE_S,Cox_ between different scenarios about heterogeneous exposure. Each scenario was simulated 1,000 times with the same sequence of random number seeds to minimize differences caused by stochastic effects.
(EPS)

S6 Fig Distribution of exposures within the vaccine arm of the trial under different scenarios about heterogeneous exposure with high transmission intensity. The height of the bars represents the relative frequency of the number of exposures in the vaccine arm for a specific realization of the virtual trial. The four simulations used the same random number seed to increase comparability among simulations.
(EPS)
