# Supplementary material for: Biased efficacy estimates in phase-III dengue vaccine trials due to heterogeneous exposure and differential detectability of primary infections across trial arms
Source: PLoS One. 2019 Jan 25;14(1):e0210041. doi: 10.1371/journal.pone.0210041 (PMC6347271; doi:10.1371/journal.pone.0210041)
Supplement: S1 Text — (DOCX) [file pone.0210041.s001.docx]

**Supplementary Text S1. Statistical estimation of sample size.**

We calculated the number of person-years in each arm of the trial based on incidence rates and on proportional hazards. We calculated the sample size of the trial necessary to estimate a VE_S_ of 0.8 with a statistical power of 0.9 (*z_2_*) and a significance level of 0.05 (*z_1_*) [33]. Based on incidence rates, the required number of person-years *y* to detect a minimum VE_S_ against the primary clinical endpoint of 0.3 was calculated as

$y=(z_{1}+z_{2})^{2}\left( \frac{1/r_{p}+1/r_{v}}{{[\ln(RR/{RR}_{L})]}^{2}} \right)$, (S1)


where RR represents the relative risk of the trial endpoint for the vaccine arm compared to the placebo arm (RR = r_v_ / r_p_) and $\mathrm{RR}_{L}$ represents the relative risk in the vaccine arm at the lower limit of efficacy that is desired (R_L_ = 1 – VE_S,min_). This ensured a high probability that the lower bound of the confidence interval excluded RR_L_. Based on proportional hazards, we calculated the total number of participants as

$N=\frac{1}{p_{v} p_{p} p_{E}}\times\left( \frac{z_{1}+z_{2}}{\log\left( 1-VE_{target} \right)} \right)^{2}$, (S2)


where p_v_ and p_p_ represent the proportion of participants in the vaccine and placebo arm, respectively, and p_E_ the overall probability of the event occurring during the trial period.

To calculate the sample size from the virtual phase-III trial, we needed an estimate of the annual incidence rate within the study period (r_p_). In field trials, this is often calculated from historic reports or field studies performed before the trial. Because incidence over the course of a trial (especially a single-site trial) can often differ from incidence estimated under either of those two assumptions, we took additional steps to ensure that our analyses would be properly powered so that we could isolate effects of heterogeneous exposure to the greatest extent possible in our analyses. To do so, we calculated the sample size of the trial based on the average r_p_ obtained from several sets of simulations of the model in the trial period (mid 2009 to mid 2010). First, from 1,000 simulations of the model, we calculated the true incidence rate of symptomatic disease during the trial period, which was on average 0.103 in children and 0.083 in adults. We then performed simulations of the trial surveillance protocol to determine that, on average, 46% of episodes of symptomatic disease among trial participants would be detected, due to underreporting and false-negative PCR tests. Thus, among trial participants, we expected r_p_ to be 0.047 among children and 0.038 among adults.

With a dropout rate of 0.1 and 12 months of follow-up, we applied eqn. S1 to obtain a required sample size of 2,324 people distributed evenly across the two arms of the trial. This implied that at least 68 cases that meet the clinical criteria associated with the primary endpoint were needed to attain 0.9 power. Based on eqn. S2, we calculated the total number of participants in the trial distributed equally between both arms (p_p_ = p_v_ = 0.5). The overall probability of a dengue case was represented as the sum of the incidence rates in each arm (p_E_ = r_v +_ r_p_). With a dropout rate of 0.1 and 12 months of follow-up, the number of participants was calculated as 2,074. The sample size was similar for both approaches. To ensure a statistical power of 90% from both approaches, we set the number of participants to the largest of both calculations (2,324). This sample size is small compared to other field trials because Iquitos experienced invasion of DENV-4 during the simulation trial period and because we were able to anticipate the number of cases during the trial with complete prescience, which would be unfeasible in a real field trial. Although uncertainty about attack rates during a trial is of major importance for trial planning, we purposefully removed this source of uncertainty to focus on our driving questions about heterogeneous exposure.
