# Supplementary material for: Biased efficacy estimates in phase-III dengue vaccine trials due to heterogeneous exposure and differential detectability of primary infections across trial arms
Source: PLoS One. 2019 Jan 25;14(1):e0210041. doi: 10.1371/journal.pone.0210041 (PMC6347271; doi:10.1371/journal.pone.0210041)
Supplement: S2 Text — (DOCX) [file pone.0210041.s002.docx]

**Supplementary Text S2. Agent-based model of dengue virus transmission.**

An ABM consists of “agents” (in this case, people and mosquitoes) who interact with each other in a shared environment. The environment of our model was represented by climate conditions and a set of locations, such as houses, schools, parks, cemeteries, and churches. Climate conditions affected mosquito biting frequency, survival, and incubation time of DENV. Locations included structures with geographic characteristics that represent the central portion of Iquitos, where prior data collection was most intense and where vaccine trials would be most likely to be conducted. Agents represented approximately 200,000 individuals that live in this area in 38,835 households, which were recorded in a geographic information system [42,43].

Our synthetic population realistically portrayed the population of Iquitos in terms of demographic characteristics of how people are distributed across houses and over time. Specifically, the demographic profiles of the modeled households were consistent with survey data collected during a previous study [11]. The population-wide sex and age distributions were consistent with U.N. estimates for Peru. To represent population changes in time, we simulated human births and deaths that match those estimated by the U.N. for Iquitos, while simultaneously preserving realistic household compositions by placing newborn children in houses with appropriately aged mothers as determined by U.N. estimates of age-specific fertility of Peru [42]. For each person, we simulated daily human movement patterns with a model previously described by Perkins et al. [16], which was fitted to data from retrospective, semi-structured interviews of residents of Iquitos [44,45].

Adult mosquitoes emerged from their aquatic development phase at different locations and immediately sought humans on whom to blood-feed. We calculated daily emergence rates with a combination of empirically derived estimates of temperature-dependent adult mosquito death rates [49] and spatiotemporal estimates of *Ae. aegypti* density in Iquitos [50]. Adult mosquitoes blood-fed on humans who were co-located with a mosquito during its time of blood-feeding at rates informed by empirical relationships with temperature [51,52]. Each day, mosquitoes moved to adjacent locations based on a probability of 0.3, consistent with another agent-based model of *Ae. aegypti* population dynamics in Iquitos [48].

The transmission of DENV to humans, and to mosquitoes, occurred through mosquito bites. The probability of transmission from humans to mosquitoes was determined by the viremia levels of the infecting human at the time of the bite [56]. After completion of a temperature-dependent extrinsic incubation period [55], infectious mosquitoes transmitted DENV to susceptible humans with a fixed probability of 1.0 [54]. Infected humans became infectious and, with a probability informed by empirical studies [63], developed symptoms following a latency period linked to the timing of peak viremia [55]. After recovering from infection, humans gained permanent immunity to the infecting serotype and temporary immunity against heterotypic infections for a period of time. The duration of temporary immunity was exponentially distributed across people with a mean of 686 days, as estimated by a previous model-based analyses of serotype-specific dengue incidence time series [57]. We assigned the initial level of population immunity to each serotype in the population based on estimates by Reiner et al. [27].
