# Supplementary figures and images for: Biased efficacy estimates in phase-III dengue vaccine trials due to heterogeneous exposure and differential detectability of primary infections across trial arms
Source: PLoS One. 2019 Jan 25;14(1):e0210041. doi: 10.1371/journal.pone.0210041 (PMC6347271; doi:10.1371/journal.pone.0210041)

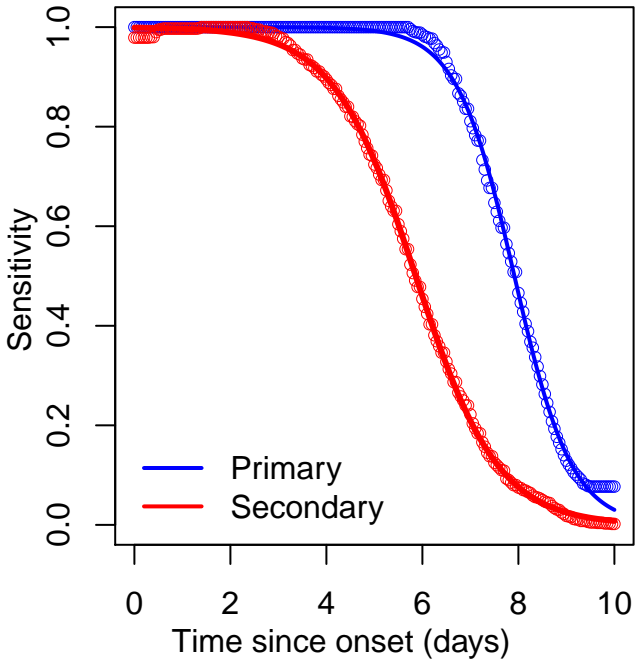

Supplement: S1 Fig — Dots represent the sensitivity curves obtained from 3,000 simulations of viremia. Solid lines represent the fitted curves described in Eq 1. For primary infections, β11 = 13.18506 and β21 = -1.665468. For post-primary infections, β12 = 6.834631 and β22 = -1.166282. (PDF) [file pone.0210041.s003.pdf]
